# Supplementary material for: Are carbon emissions trading and green financial instruments synergistic? -Comprehensive quantitative research based on content analysis
Source: PLoS One. 2024 Mar 7;19(3):e0298601. doi: 10.1371/journal.pone.0298601 (PMC10919601; doi:10.1371/journal.pone.0298601)
Supplement: S1 Data — (ZIP) [file pone.0298601.s001.zip › Data/地级市Z_F工作报告.zip!/╣π╬≈/┴°╓▌╩╨/└┤╫╘╣1⁄2╓┌║┼í╛┬φ┐╦╩2╛▌═°í┐_2009.docx]

各位代表：

现在，我代表市人民政府向大会报告工作，请予审议，并请各位政协委员和列席人员提出意见。

2008年工作回顾

过去一年，是非同寻常、极不平凡的一年。这一年，吴邦国委员长亲临柳州视察指导，给我们带来了亲切关怀和巨大鼓舞。这一年，我们以自治区成立50周年大庆为契机，在市委的正确领导下，坚持以科学发展观统领全局，紧紧围绕发展和民生两大主题，继续解放思想，优化发展环境，扎实推进“三个同步”，全面实施“二次创业”，圆满完成了市十二届人大五次会议确定的目标任务。

过去一年，也是柳州人民攻坚克难、团结奋进的一年。面对年初历史罕见的雨雪冰冻灾害和年中的严重洪涝灾害，我们紧紧团结和依靠全市人民，上下一心，众志成城，取得了抗击雨雪冰冻灾害和抗洪救灾的重大胜利；面对四季度突如其来的国际金融危机，我们坚决和企业一起，积极行动，沉着应对，保持了经济的平稳较快发展和社会的和谐稳定。

初步统计，全市实现地区生产总值900.6亿元，增长13.2%；工业总产值1750亿元，增长25.9%；财政收入140.1亿元，增长20.4%；全社会固定资产投资430.3亿元，增长42.5%。单位生产总值能耗下降5%。城镇居民人均可支配收入14350元，增长11.5%；农民人均纯收入3855元，增长10.2%。城镇登记失业率控制在4.3%以内。人口自然增长率控制在自治区下达指标以内。为民办20件实事基本完成。

一、工业发展态势稳定，结构调整和产业升级步伐加快

工业发展实现“六个百亿”①新突破。全市完成规模以上工业产值1455亿元、主营业务收入1413.4亿元，分别增长26.7%和22.8%；实现利润总额41.1亿元，下降41.5%。三大支柱产业、五大传统优势产业分别实现产值过千亿和过百亿目标。全市汽车产销突破70万辆。“六个百亿”全面实现，即柳钢、上汽通用五菱、柳工主营业务收入分别迈上300亿元、200亿元、100亿元台阶，鹿寨、柳南、阳和工业新区工业产值均突破百亿元大关。

工业投入明显加大。完成工业投资158亿元，增长35.1%；其中更新改造投资140亿元，增长39.6%。柳钢冷轧板带项目竣工投产，柳工自主研发的中国最大轮式装载机成功下线，柳汽“风行景逸”新车型顺利上市。一批重点技改项目的实施和中小企业技改力度的加大，进一步增强了工业发展后劲。

节能降耗取得重大进展。单位生产总值能耗下降5%。41户重点能耗企业节能90万吨标准煤，提前两年完成“十一五”目标。循环经济有序开展，柳钢、鱼峰水泥、柳化成为自治区级循环经济试点企业，柳化、川东磷化节能技改列入国家财政奖励节能项目。

园区开发建设加快推进。出台关于加快柳东新区建设的政策性文件，进一步理顺了新区管理体制。柳东新区完成投资45.2亿元，增长47.2%。重点监测的六个工业园区②规模以上工业产值337.8亿元，增长55.1%。信息产业园正式开园，洛维工业集中区动工建设；柳南河西工业区、柳北工业区通过自治区A类园区认证，自治区级工业园区增至6个。

自主创新能力持续增强。第四轮创新计划全面实施，新增4家自治区级工程技术研究中心；有15个项目获广西科技进步奖，13个创新项目纳入国家重点新产品或科技支撑计划。“双百人才”工程深入实施，人才小高地建设扎实推进，新增2家企业博士后工作站。

应对金融危机出手快、措施实、力度大。面对日益加剧的国际金融危机，迅速推出安排7000万元财政专项资金支持中小企业技术改造和技术创新、协调金融机构安排30亿元信贷额度并按国家基准利率扩大中小企业贷款规模、将中小企业担保公司年担保费率从1.92%下调到1%、安排500万元职工岗位技能培训专项资金等十大措施，与企业一道，坚定信心，抢抓机遇，共渡难关。全年市财政用于扶持企业发展资金达2.2亿元。新增市中小企业担保公司资本金8000万元，提供担保16亿元，增长58.4%；其他民营担保公司共为中小企业提供担保15亿元。落实搬迁改造政策，佳力电工等6家市区企业启动搬迁改造。积极开展“中小企业服务月”活动，从9月起实行每月10日市长“企业接待日”制度，帮助企业解决困难和问题。

二、“三农”状况持续改善，社会主义新农村建设扎实推进

农业产业结构进一步优化。粮食生产稳中有升，蔬菜和甘蔗种植面积有较大增长，特色农产品基地建设加快推进。农业产业化进程加快，新增自治区级重点龙头企业2家、市级8家，新增农民专业合作社61家。扶持养殖业发展初见成效，新建规模以上禽畜养殖场280个。“千村两机”工程③深入推进，农机购置补贴政策全面落实。

农村基础设施持续改善。建成通乡、通村公路887公里，基本实现乡乡通油路，90.9%行政村通公路。恢复改善农田灌溉面积7万亩，完成病险水库除险加固15座，建成农村饮水安全工程80处，解决了8.2万人口饮水问题。完成造林28.3万亩。新建沼气池8015座，改厕8500座；改造农村特困户茅草房3530户。积极开展村庄整治，61个新农村建设试点村屯基础设施状况明显改善。第二批85个贫困村整村推进扶贫开发任务如期完成。

县域经济蓬勃发展。六县实现地区生产总值288.6亿元，增长14.7%；财政收入19.3亿元，增长10.5%；全社会固定资产投资149.3亿元，增长36.3%。县域工业增势强劲，六县规模以上工业总产值216.2亿元，增长30.6%。柳州鑫能秸秆发电、柳化40万吨二甲醚等重大项目相继落户柳城、鹿寨等县，进一步增强了县域工业发展后劲。

三、服务业加快发展，城市服务功能不断增强

商贸流通业加快发展。出台了关于加快服务业发展的三个政策性文件，加大对服务业发展的扶持和引导力度。全年实现社会消费品零售总额335亿元，增长22.2%。龙城路地下商业街、东环新市场竣工使用，桂中海迅柳北物流配送中心一期加快推进。广西糖网、工贸、百货、尚龙电器等大型商贸企业快速发展。“万村千乡市场工程”④和“双百市场工程”⑤稳步推进。着力加强民生价格监控，有效遏制了物价过快上涨的势头。

旅游业快速发展。旅游资源整合和项目开发力度加大，“百里柳江”、“百里风情”和“环都市乡村旅游”三大旅游产品集群初现雏形。三江程阳、融水贝江分别通过4A、3A景区验收。成功举办第二届国际小丑狂欢节和第五届柳州国际奇石节。全年接待国内外旅游者908万人次，增长15.6%。

房地产业持续健康发展。积极应对国际金融危机，及时推出增强房地产企业资金流动性、允许企业分期缴纳土地出让金、放宽公积金住房贷款政策等14项措施，稳定了房地产市场。房地产业完成投资102.5亿元，增长39.1%。

四、城市建设管理取得新成效，城市形象明显提升

城市基础设施进一步完善。基础设施重大项目竣工20项，新开工71项，完成投资67亿元，增长37%。外环路建设全面启动。静兰桥改建完成，柳太路、柳长路南段顺利竣工，西江路口、鱼峰路口下穿通道及北雀路改造等一批项目投入使用，重点路段交通压力得到缓解。拓展对外大交通取得重大进展，柳肇铁路纳入国家建设规划，鹿寨至阳朔、三江至桂林、三江至北海高速公路及柳江航道疏浚相关工作加紧推进。柳东新区道路、管网等基础设施不断完善。旧城改造稳步推进，水南、静兰片区城中村改造启动。久悬未决的殡仪馆搬迁问题得到解决，新馆建设进展顺利。

城市人居环境进一步改善。生态环保治理卓有成效。第一次全国污染源普查顺利完成。所有国控重点污染源在线监测实现联网。关闭了对机场周边空气污染严重的白莲淀粉厂，制糖、造纸行业的一批污染治理工程顺利完成。全面推进城镇污水生活垃圾处理设施建设，龙泉山（二期）和白沙污水处理工程投入试运行。三中干渠、冶炼干渠等内涝整治工程顺利完工。噪声扰民综合治理得到加强。柳江饮用水保护河段保持国家地表水Ⅲ类水质标准；市区空气质量优良天数达359天，优良率98.4%。

城市形象进一步提升。积极创建“国家园林城市”，“百万人民种百万棵树”活动成效明显，新增城市绿地182.1公顷，城市绿地率达32%、绿化覆盖率37%、人均公共绿地面积10平方米，三大指标已达到国家园林城市标准。园林及景观建设成效明显，蜡烛山公园、河东公园等七个公园完成建设改造，蟠龙山瀑布群改造、滨江两岸景观改造、小街小巷改造和“穿衣戴帽”工程、夜景亮化一期工程顺利完工。

城市规划和管理水平进一步提高。城市规划和土地管理工作得到加强，建设用地保障能力不断提高。国内一流的城市规划展览馆落成开馆，激发了市民关注城市、热爱家乡的热情。创建文明城市、争创“南珠杯”活动深入开展，“城乡清洁工程”和“五项整治”纵深推进。垃圾处置日趋规范，在全区率先实现建筑垃圾密闭化运输，市区医疗垃圾实现无害化处理。“数字城管”建设稳步推进。在“百里柳江”推行水行政综合执法工作，柳江河面违法行为整治力度加大。

五、民生持续改善，社会保持和谐稳定

就业再就业工作取得新进展。全面贯彻《就业促进法》，制定出台12个促进就业和创业的配套政策。全年城镇新增就业人数4.6万人，其中下岗失业人员再就业1.4万人，就业困难对象再就业4355人。全市农村新增转移就业4.8万人，转移就业培训3.2万人。面对国际金融危机，出台专门政策，给返乡农民工提供３个月免费培训，期间给予每人每月300元的补贴。

社会保障工作进一步加强。提高了企业离退休人员基本养老金标准和农村低保月补差标准。从降低就诊起付标准和个人支付比例等9个方面对医保政策进行了调整。出台了被征地农民培训就业和社会保障、餐饮服务业农民工参加工伤保险等政策。加强保障性住房建设，全年竣工经济适用房11.2万平方米，新增廉租房保障对象1600户。“阳光计划”深入推进，建成残疾人康复中心，改造农村贫困残疾人危房800户，历经三年全面完成了农村贫困残疾人危房改造任务。救灾救济工作有效开展，雨雪冰冻灾害和“6•13”洪涝灾害倒塌民房恢复重建全部完成，7734户受灾群众搬入新居。在“5•12”四川汶川大地震中，全市共筹到赈灾资金6333万元，并出色完成了灾区伤员救治和支援灾区任务。

三大民生工程顺利推进。一是在全区首创改制企业职工危旧房集中区改造工程，力争用三年时间基本完成改造任务，惠及人口近10万人。年内已启动5个改造项目，涉及19家企业3.4万人，拆迁面积68.4万平方米，拟建面积250万平方米。这一工程开创了全国旧城改造的柳州模式，得到了市民的拥护和自治区的肯定。二是在全区首创贫困县九年义务教育农村学生免费午餐制度，市、县两级财政投入4000多万元，受益学生10万人。三是实施少数民族村寨防火改造工程，首批211个村屯的防火改造全面推进。

社会大局保持稳定。启动新一轮平安柳州建设。为期三年的“三基”工程建设基本完成，得到了国家公安部的表彰。“天网工程”一期建成使用，实现了对重要路段和复杂场所的全天候监控。社会治安综合治理卓有成效，刑事案件持续下降。以“平安奥运”为契机，畅通信访渠道，加大处纠力度，积极开展“公开大接访”活动，有力维护了社会稳定。我市先后被评为2005—2007年自治区平安市、建设平安广西活动先进市，并成为广西唯一的全国科技强警示范城市。食品药品安全监管力度加大。抓好安全生产隐患排查治理，安全生产基层基础建设进一步加强。

六、改革开放取得新成绩，招商引资成效显著

经济体制改革不断深化。国有企业战略重组取得新进展，中国有色矿业集团对柳锌资产进行并购重组；柳江纸厂增资扩股、柳化战略性重组、工贸与百货公司合并重组等项目加快推进。投融资体制改革取得重大突破，组建了产业、基础建设与公共事业、金融与担保三大类多元化投融资平台，全年实现融资68.5亿元，并首次采用BT模式⑥融资27亿元建设“三桥一路”。金融生态环境不断改善，浦发、光大、邮政储蓄银行及瑞达期货等相继入驻柳州。金融机构年末存款余额852.3亿元，贷款余额564.0亿元，分别比年初增长17.5%和23.9%。

对外开放进一步扩大。全市进出口总值20.7亿美元，增长52.6%；其中出口9.6亿美元，增长37.7%。实际利用外资1.05亿美元，增长27.4%。“三结合三为主”的招商新模式取得明显成效，全年签约项目424个，合作金额310亿元，增长76.7%；市外境内到位资金250.3亿元，增长60.6%。承接东部产业转移企业148家。中国重汽成为第四家在柳设立生产基地的国内大型汽车集团。中航工业、金城集团以增资扩股方式与方盛实业共同组建乘龙专用车有限公司。

七、社会各项事业全面发展，群众精神文化生活更加丰富

教育事业稳步发展。“两基”继续得到巩固，“普九”水平不断提高。推进城市义务教育段学生免收杂费工作，受益学生9.6万人。接收外来务工子女就学5.4万人。普通高中规模发展稳步推进，高考成绩继续保持全区领先。全面推进职教攻坚，市财政职教攻坚经费达1.9亿元，职教园一期工程正式启动建设。

文化、体育事业加快发展。文化致富工程加快推进，建成国家文化信息资源共享工程支中心2个，城乡文化惠民工程活动点105个，农家书屋185家。“柳江之夏”、龙狮争霸赛等群众文化活动蓬勃开展。“扫黄打非”行动深入推进，文化市场环境明显改善。第三次全国文物普查全面开展。历史文物和非物质文化遗产保护得到加强，有8个项目列入第二批自治区级非遗名录，市博物馆荣获全国文物保护奖。文化产业项目建设加快推进。全民健身活动蓬勃开展。李宁点燃北京奥运会圣火，柳州籍运动员陆永和江钰源勇夺奥运金牌。成功举办F1摩托艇世界锦标赛中国柳州大奖赛，提升了柳州的国际知名度。

卫生及人口与计生工作取得新进展。市人民医院新院和市疾控中心建成使用。农村卫生工作不断加强，新建甲级村卫生所200所，建设改造乡镇卫生院22个。社区卫生服务设施继续完善，我市被确定为国家社区卫生服务体系重点联系城市。新型农村合作医疗全面铺开，参合农民208.9万人，参合率达88.5%。人口与计划生育工作新机制初步建立，低生育水平进一步稳定，全市符合政策生育率达89.8%。

精神文明建设不断加强。思想道德建设扎实推进，妇女儿童发展纲要全面落实。我市获首批“全国未成年人思想道德建设工作先进城市”称号。精神文明创建活动深入开展，荣获“全国创建文明城市工作先进城市”称号；鹿寨县被评为“国家卫生县城”。总工会大楼开工建设。国防教育有效开展，双拥共建不断加强。认真组织开展自治区成立50周年大庆活动，全面落实民族政策，少数民族地区发展加快，各民族团结和睦。

八、政府工作不断改进，发展环境进一步优化

解放思想和学习实践科学发展观取得新成果。通过继续解放思想大讨论，开阔思路，凝聚共识，改进工作，进一步营造了良好的发展氛围。深入开展学习实践科学发展观试点活动，结合自治区“科学发展三年计划”，明确了“十一五”期后三年的工作目标，在全市掀起了全面实施“二次创业”、建设“四个名城”的高潮。

政务环境不断优化。深化行政审批制度改革，在前四次集中清理的基础上，再压缩审批事项145项，下放县区142项，进一步提高了行政审批效率。开展“二次创业”绩效考评，强化落实和督促检查，行政效能建设明显改进。经济普查工作顺利开展。理顺城区财政管理体制和城市建设管理权限，进一步提高了城区的城市管理水平。

民主法治和党风廉政建设得到加强。市政府自觉接受市人大、市政协的监督，广泛听取社会各界的意见和建议，认真办理人大代表议案、建议和政协委员提案，答复率均为100%。“五五”普法全面开展，依法治市扎实推进。基层民主政治建设不断加强，第五届村委会和第三届社区居委会换届选举圆满完成。党风廉政建设责任制全面落实，政风行风建设取得新的成效。加强对市直行政事业单位国有资产收益管理，对近千处行政事业单位国有房产租赁权首次进行了公开拍卖和市场化运作，租金收入增长52%。

一年来，我市人事、编制、外事侨务、金融、工商、税务、技术监督、统计、审计、国家安全、决策咨询、档案、人防、气象、粮食、农机、宗教、老龄、修志、防汛抗旱、防震减灾等工作都取得了新的成绩。

各位代表，回顾2008年工作，成绩来之相当不易。这是市委正确领导及市人大、市政协监督支持的结果，是全市人民团结奋斗和社会各界大力支持的结果。在此，我代表市人民政府，向全市人民，向驻柳人民解放军、武警部队官兵，向各民主党派、工商联、人民团体及海内外所有关心和支持我市发展的各界人士，致以崇高的敬意和衷心的感谢！

在总结成绩的同时，我们也清醒地看到了存在的困难和问题。一方面，国际金融危机影响日渐加剧，规模以上工业企业有三成以上主营业务收入同比下降、五成以上企业利润同比下降。另一方面，一些深层次或带有普遍性的问题仍没有得到很好解决，比如工业结构不尽合理，一些中小企业管理粗放，创新能力不强，缺乏自主知识产权的品牌和技术，抵御市场风险能力较弱；土地、资金瓶颈制约明显；城乡发展不够协调；农民增收办法不多；环境保护压力较大；就业形势不容乐观；民生状况亟待改善等等。对这些问题，我们要认真研究，积极应对，努力解决。

2009年工作任务

今年将是近十年来我市经济最为困难的一年。当前，国际金融危机持续蔓延，国内外经济形势异常严峻。金融危机对我市经济的冲击已全面显现，经济增速明显回落，工业利润大幅下降，外贸出口萎缩，消费热点降温，就业压力加重，农民增收困难，经济运行的难度越来越大。今年也是蕴含重大机遇的一年。中央实行积极的财政政策和适度宽松的货币政策，以强有力的措施应对金融危机，以罕见的力度拉动内需、促进增长，这将对以投资拉动为主的柳州经济带来极为难得的发展机遇。面对新的形势和任务，我们必须增强危机意识、忧患意识、机遇意识，以非常办法、非常措施、非常力度、非常政策，趋利避害，抢抓机遇，突破困境，加快发展，确保今年地区生产总值突破1000亿元、工业总产值突破2000亿元，再次实现工业总产值三年翻一番。

今年政府工作的总体要求是：坚持以科学发展观统领全局，深入贯彻市委提出的“科学发展，城乡统筹；经济突围，项目突破；全民奋勇创业，共建和谐柳州”二十八字方针，扎实推进“三个同步”，全面开展“二次创业”，紧紧围绕“经济发展突围年”、“项目建设突破年”、“全民创业突进年”和“民生改善突出年”，以保增长为首要任务，扩投资、扩市场、扩消费，努力提升经济质量、提升创新能力、提升创业环境、提升城市品位、提升民生福祉，推动“经济升级，城市转型”，确保经济平稳健康较快发展及社会和谐稳定。

今年我市经济和社会发展的主要预期目标是：地区生产总值增长11%；工业总产值增长14.3%；财政收入增长8%；全社会固定资产投资增长25%；单位生产总值能耗下降5%以上；主要污染物二氧化硫、化学需氧量排放分别减少2.5%和4.1%；城镇居民人均可支配收入、农民人均纯收入分别增长10%；城镇登记失业率控制在4.5%以内；人口自然增长率控制在8‰以内。

为实现上述预期目标，着重抓好九个方面的工作：

一、采取措施促进工业稳定增长，加快工业结构调整

紧紧围绕“经济发展突围年”，加大扶持引导力度，积极应对国际金融危机，确保工业经济平稳运行，促进工业结构调整，加快“工业名城”建设。

千方百计帮助企业摆脱困境，确保工业总产值突破2000亿元。全面落实我市扶持中小企业应对金融危机十大措施，落实国家当前缓缴半年社保费用、降低四项社保费率等应对金融危机措施，并根据形势变化适时推出相应政策措施，全力帮助中小企业渡过难关。鼓励和支持三大支柱产业加快发展，进一步提高本地配套率，带动中小企业冲出困境。号召和引导企业加强形势分析和市场研究，及时制定新的生产经营战略，加大新产品研发力度，抢市场，清库存，降成本，调结构，引导企业不减员，努力保持企业正常生产经营。着力帮助企业解决资金难问题，设立风险投资资金，建立国有资本择进适退运作机制，加快产业投融资平台建设，加大融资担保力度，极力营造更加良好的金融生态环境。强化协调服务，优化发展环境，继续开展“中小企业服务月”活动，坚持市长“企业接待日”制度；切实做好税收指导、培训和服务工作，确保企业充分享受到税制改革的实惠。

实施项目带动战略，加快产业结构调整。鼓励和支持企业抓住国家增值税转型机遇，加大技改投入。今年工业投资要达到150亿元以上，其中更新改造投资140亿元以上。重点推进柳钢热轧板带二期、上汽通用五菱轿车、五菱专用车生产基地、柳化20万吨烧碱和20万吨聚氯乙烯等一批重大项目建设，进一步做大做强支柱产业，改造提升传统优势产业，培育发展生物制药、机电仪一体化等新兴产业以及化工、林浆纸一体化、铝业等战略产业。进一步扩大循环经济试点，积极争创国家循环经济试点城市。

加强自主创新，加快发展方式转变。今年市财政安排4000万元专项资金，支持企业开展产品创新和技术创新。深入实施第四轮创新计划，进一步完善自主创新体系和中小企业公共服务平台；积极推进区域性质量技术监督检测基地和茧丝绸、铟、汽车零部件等产品监督检验中心建设，为企业自主创新提供良好条件。实施技术标准战略。发挥企业技术领先优势，引导企业参与国家标准、行业标准的制定。积极推进第三批企业专利试点。鼓励和支持企业争创名牌，年内争创2个以上中国名牌产品或驰名商标。高度重视人才的培养和引进，实施“企业家成长促进计划”和“高技能人才培养工程”，打造一批高素质的企业家和技术人才队伍。

加快新区和工业园区建设，大力发展园区经济。突出抓好柳东新区开发建设，进一步完善阳和、官塘、古亭片区道路、排水管网、标准厂房、商务办公等基础设施，着重抓好工业品展示交易中心、柳东大厦、阳和企业服务中心等项目建设，全力推进乘龙专用车、铝深加工等一批重大产业项目，确保新区完成投资58亿元以上。继续创新园区管理体制，不断完善园区基础设施，增强园区的产业集聚能力。今年，重点监测的六个工业园区工业产值要达到420亿元以上。

二、大力开展“项目建设突破年”活动，全力推进重大项目建设

紧紧围绕国家投资的方向和重点，全力以赴抓投资，千方百计上项目，以项目突破带动经济突围。今年，全社会固定资产投资要达到540亿元以上。

全力推进重大项目建设。实施重大项目287项，涉及总投资889亿元，今年计划投资245亿元。一是围绕增强产业发展后劲和促进产业升级，突出抓好101项重大产业项目，年度投资73亿元以上。其中重大工业项目39项，年度投资44亿元以上。二是围绕完善城市功能、提升城市形象和巩固交通枢纽地位，实施115项城市建设重大项目，年度投资105亿元以上。三是围绕水利交通、电网建设、村寨防火、环保设施和社会主义新农村建设五大方面，实施25项统筹城乡发展重大项目，年度投资32亿元以上。其中，电网建设和改造确保完成投资20亿元以上。四是围绕医、学、住、行等事关人民群众切身利益问题，切实抓好46项重大民生和社会项目建设，年度投资35亿元以上。

建立健全重大项目建设保障机制。一是建立投融资保障机制。积极争取国家、自治区资金支持，努力扩大投融资平台融资规模，尽力促成银行增加信贷额度，以及通过招商引资、发行债券、企业上市等多种途径，拓宽融资渠道。二是建立项目建设用地保障机制。坚持集约节约利用土地，依法推进征地拆迁，加强土地储备运作，加大闲置土地处置力度，努力保障建设用地。三是建立项目建设推进机制。继续实行重点项目领导联系制度和重点项目联审制度，开辟重大项目建设绿色通道，确保项目建设各个环节畅通无阻。四是建立重大项目储备机制。根据我市发展需要，抓紧策划一批重大项目，切实做好项目前期工作，增强项目储备。五是建立健全督查审计机制和奖惩机制，确保项目建设进度和质量，严肃查处违法违规和徇私腐败行为。

三、实施城乡统筹发展新格局战略，加快城乡一体化进程

全面贯彻落实党的十七届三中全会精神，建立以城带乡、以工促农、以商强农的城乡统筹发展的新体制和新机制，扎实推进社会主义新农村建设，加快形成城乡经济社会发展一体化新格局。

加强规划引导和产业带动，统筹城乡协调发展。统一编制城乡建设发展规划，力争用两年的时间完成乡镇和村屯规划编制；启动柳江县撤县建区工作；加快推进乡镇基础设施建设，完成一批新农村试点建设及村庄整治项目。统筹城乡产业布局，推进柳州-新兴-进德-拉堡、柳州-西鹅-拉堡、柳州-雒容-鹿寨、柳州-沙埔-柳城沿路经济带建设，分别形成城南、城东、城北经济带，把柳江、鹿寨、柳城三县打造为以市区为中心的“一小时经济圈”；以建设三江至柳州的高速公路为契机，把融安、融水、三江三县打造为以市区为中心的“两小时经济带”。建立健全财政支农支出稳定增长机制。今年要拿出四分之一新增可用财力用于支农建设，以后每年要拿出一定比例新增可用财力用于支农建设。每年拿出1000万元支持六县发展种养业。

掀起农村基础设施建设新高潮。开展全市水利设施建设大会战，完成15座病险水库除险加固，建成农村人饮工程191处，解决10万人饮水安全问题。启动落久、木洞水利枢纽建设，力争洋溪水利枢纽早日开工，让柳州人民彻底解除洪灾的后顾之忧。加强农村交通基础设施建设。新建农村公路项目87个，建设里程668公里。年内新增41个行政村通等级公路，新增39个行政村通沥青（水泥）路。加大沼气能源建设和卫生改厕力度，新建沼气池10000座，改厕9000座。

大力发展现代农业。加强耕地保护，稳定粮食生产，扩大甘蔗良种种植。大力发展桑蚕、水果、蔬菜、茶叶等优势产业，推进茶油、毛竹低改，努力打造一批市场竞争力较强的特色农业品牌。加强养殖基地建设，推进规模养殖发展。加快农业产业化进程，扩大农业适度规模经营，积极推进柳南“金太阳”⑦、柳北长塘青茅花卉基地等生态农业示范项目建设；扶持壮大一批农业产业化龙头企业，力争新增市、区级重点龙头企业10户以上。大力发展农民专业合作社，充实完善农业科技队伍，推行村村派驻科技特派员制度，不断加大农业技术推广、动植物疫病防控、农产品质量监管等公共服务力度。全面实施“千村两机”工程和“千乡万村现代农机装备推进工程”⑧，努力提升农业生产机械化水平。

四、提升商贸流通业发展水平，推动服务业全面升级

紧紧围绕“经济发展突围年”，努力扩大消费需求，全面加快服务业发展，有效拉动经济增长。今年全市社会消费品零售总额要达到400亿元，增长19.4%。

积极扩大城乡居民消费。落实和完善促进房地产开发和住房消费的政策措施，引导合理的房地产投资和住房消费，推动房地产业健康稳定发展。加大扶持引导力度，积极促进汽车、钢铁、机械、建材等本地产品消费。继续实施“万村千乡市场工程”，加快完善农产品流通网络和农业生产资料流通体系，全面推进家电下乡、农机下乡工作，鼓励和支持企业开发适合农村市场消费的机电产品，努力拉动农村消费。加强市场调控和监管，切实改善市场环境，促进安全消费。

大力发展商贸流通业。加快商贸物流基础设施建设，大力推进钢材物流园、铁路集装箱物流园、河西工业物流园、胜利大超市等重点商贸物流项目；加快汽车贸易园二期建设和柳邕农副产品市场、粮食批发市场搬迁改造，推进农贸市场升级改造。不断完善河东片区、柳东新区商业设施，积极培育城市区域性商业中心。培育大型流通企业集团，促进中小商贸企业发展。大力引进国内外知名商业企业，推进百货、工贸合并重组和桂中海迅整合资源、增资扩股，努力把桂中海迅、广西糖网培育成为国家级品牌企业。

加快建设旅游名城。大力推进旅游重点项目建设，完成全市旅游总体规划修编和重点景区开发规划的编制，推进“百里柳江”游船项目的实施，启动“百里柳江”标志性景观建设，积极开展“金钉子”⑨项目前期工作；深度开发三江程阳等“百里风情”旅游资源，丰富民族风情旅游文化内涵。推进市博物馆、鹿寨香桥、融水贝江和三江丹洲创建国家4A级景区工作；大力发展乡村旅游，加快建设“环都市农业观光休闲旅游圈”，创建一批全国及自治区农业旅游示范点。加大旅游宣传力度，加强旅游区域合作，深度推进柳州--桂林旅游一体化。

加快发展新型服务业。以产业为依托，推动生产性服务业发展，促进现代服务业与先进制造业的融合。加快区域性金融中心建设，积极引进各类金融保险机构，推进地方金融机构的改革和发展，进一步增强金融对地方建设发展的支持力度。大力发展会展、连锁经营、电子商务等新兴业态，做大做强广西铟谷有色金属交易中心。鼓励发展各类中介服务业，努力满足多样化的社会服务需求。完善社区便民服务设施，大力发展家政服务、便民早餐等社区服务，使之成为就业的新增长点。

五、掀起新一轮的大规模城市建设，打造现代宜居城市

紧紧围绕“项目建设突破年”，坚持以人为本，加大城市规划、建设和管理力度，着力提高城市的聚集和综合承载能力，不断改善人居环境，加快建设现代宜居城市。

以建设“十大工程”为重点，再掀城市建设新高潮。新开工基础设施重大项目49项，续建53项，竣工13项。一是全面推进城市快速环道、三座跨江大桥、风情港、金沙角、李宁体育馆、游泳馆、环江滨水大道、职教园、地王财富中心、窑埠古镇等十大工程建设，着力打造一批城市建设精品。二是积极拓展对外大交通。成立柳肇铁路办公室和柳江航运办公室，全力促成柳肇铁路年内开工建设，加快柳江航道疏浚及码头建设，积极配合自治区打造“西江亿吨黄金水道”。协助做好贵广高速铁路柳州段建设工作，大力推进鹿寨至阳朔、三江至桂林、三江至北海高速公路建设，实施柳州机场改扩建工程。三是全面加快城市进出口道路和中心路网建设。着力推进柳工大道、柳邕路、东环路延长线等34条城市道路的建设和改造。注重将路桥建设改造与彰显城市山水景观相结合，精心打造集交通、运动、休闲等功能为一体的环江滨水大道，在城市主干道车流人流集中区规划建设一批车辆下穿通道。四是大力推进旧城改造。继续实施“穿衣戴帽”工程和夜景亮化工程。积极推进城中村、城郊村整体改造，重点推进静兰片区、古亭片区整体改造，打造现代城市片区典范。加快殡仪馆新馆建设，确保上半年完成殡仪馆搬迁。

深入实施“碧水蓝天，青山绿地”工程，进一步改善城市生态环境。今年要力争荣获“国家园林城市”，全力夺取“中国人居环境范例奖”，全面启动“国家环保模范城市”及“节水型城市”创建工作。一是突出抓好园林绿化。年内完成华丰湾堤、龙潭路、北雀广场等一批绿地景观工程；推进马鹿山奇石休闲公园、土伏山、桐油山等公园建设；新建官塘植物园，并在园中开辟荟萃世界各地柳树品种的柳园。继续抓好地质环境恢复治理，推进河岸及石山绿化。二是加强环境综合治理。开展“保护母亲河行动”，切实加强柳江河水环境治理，坚决关闭淘汰和搬迁改造市区污染企业，完成所有重点污染源在线监控系统建设，确保减排目标圆满完成。继续开展恶臭污染、噪声扰民和餐饮业油烟治理等环保专项整治行动，严厉打击环境违法行为。三是全面推进城镇污水垃圾处理设施建设。竣工阳和污水处理工程，加快柳东、沙塘片区污水处理工程和立冲沟垃圾填埋场二期建设，全面推进六县污水垃圾处理工程建设。加强林业生态体系建设，加大柳江上游水源林保护力度。要力争柳江饮用水保护河段水质达到国家Ⅱ类标准、市区空气质量全面达到国家二级标准。

提高城市规划和管理水平，巩固和提升“山清水秀地干净”的城市形象。加快推进城市总体规划调整和土地利用总体规划编制，完善城市各片区控制性详细规划，实现市区控制性详细规划覆盖率90%以上。加强规划执法和用地监督管理，严肃查处违法建设、违法用地行为，切实加大“百里柳江”两岸整治工作力度。深入推进“城乡清洁工程”，继续开展“五项整治”，着重整治农贸市场、临时市场、早市夜市的卫生环境，理顺户外广告管理，营造整洁有序的市容环境。全面加快“数字城管”建设。在“百里柳江”推进水行政综合执法工作，加大柳江河面违法行为整治力度。加强执法队伍建设，规范执法行为。

六、创新体制机制，深入推进改革开放

坚持以改革开放促进结构调整，推动经济升级。今年引进市外境内资金要达到230亿元，实际利用外资1.2亿美元，外贸出口突破10亿美元。

继续深化体制改革。一是深化经济体制改革。推进投融资体制改革，加大公共资产的划转整合力度，不断完善三大类投融资平台，积极创建县区、园区投融资平台。加快小额贷款公司的试点和推广工作。鼓励企业加大兼并重组力度，积极引进战略投资，不断提升企业综合竞争力。二是加大统筹城乡改革与发展工作力度。继续推进农村综合改革试点。深化农村集体土地使用制度改革，稳步推进农村土地经营承包权流转试点，推进城乡建设用地增减挂钩试点。稳步推进集体林权制度改革。切实抓好华侨农林场的改革和发展工作。进一步完善农村金融服务体系。

加大招商引资力度。坚持“三结合三为主”的招商思路，努力实现招商升级、服务提速。突出产业链招商、专题招商和定向招商，提高招商引资的针对性与实效性。立足优势资源、优势产业和战略产业，以资源引资产，以存量引增量，以环境引资金，加大重点领域和关键项目的招商引资力度，积极承接产业转移，努力引进世界500强、中国500强企业，推进“央企入桂”工作。强化招商引资大兑现工作，做到政府兑现承诺，企业兑现投资，切实提高项目履约率、开竣工率和资金到位率，确保项目进得来、落得下、留得住、发展好。

加快外经外贸发展。落实和完善支持外贸出口的政策措施，努力稳定传统出口市场，积极开拓新的出口市场，稳定并努力提高重点企业和传统大宗商品的出口，力争在拓展东盟市场上取得新的突破。坚持以质取胜战略，切实转变外贸增长方式，优化出口产品结构，提高工业制成品、高新技术产品出口比重。积极参与多区域合作，主动融入北部湾经济区开发建设，全面加快与东盟国家产业的对接。积极鼓励企业开展对外工程承包和国际劳务合作，进一步拓宽对外合作渠道。加强口岸基础设施建设，不断改善通关环境，提高口岸通关水平。

七、突出抓好民生建设，让人民群众充分享受改革发展成果

紧紧围绕“民生改善突出年”，抓住国家拉动内需加大民生投入的良机，把改善民生作为保增长的出发点和落脚点，全力推进民生建设。今年，要继续为民办好20件实事。

大力开展“全民创业突进年”活动，促进就业再就业。积极构建税收优惠、金融支持、用工鼓励、创业保障等全民创业政策体系，营造良好的全民创业环境，培育壮大全民创业主体，力争年内个体工商户户数增长10%，私营企业户数增长12%。贯彻落实《就业促进法》，进一步完善公共就业服务体系，不断完善面向所有困难群体的就业援助机制，确保实现“零就业家庭”动态消除的目标。认真做好大中专毕业生就业服务，建立企业招收大学毕业生的政府激励机制。加大就业培训力度，开展中小企业职工岗位技能培训和返乡农民工专项职业技能培训，重视农民工返乡就业、创业工作，促进就近转移就业。今年，要确保城镇新增就业人数4.5万人，培训农村富余劳动力3万人，实现转移就业4万人。加大劳动保障监察力度，切实维护企业员工合法权益。

进一步完善社会保障体系。根据上级统一部署，适当提高企业退休人员的养老保险金。切实抓好农民工参加养老保险和被征地农民培训就业与保障工作。继续推进城镇居民基本医疗保险试点，不断提高城镇居民的参保率。积极推进“政策保险进农业、商业保险进农村、社会保险进农家”工作。进一步提高城镇和农村低保标准，逐步提高村干部生活补贴。整合民政资源，建设社会福利救助中心，进一步完善社会福利救助体系。统筹推进城乡医疗救助，逐步建立城乡一体化的医疗救助体系。继续实施关爱残疾人“阳光计划”。加快推进居家养老试点，多层次解决养老问题。抓好扶贫开发，启动第三批贫困村整村推进扶贫开发工作。

全力推进一批重大民生工程项目。一是全面落实国家保障性住房政策，加快安居工程建设。着重抓好改制企业职工危旧房集中区改造工程和经济适用房、廉租住房建设，为32家改制企业职工实施危旧房改造70.5万平方米，新开工建设廉租房和经济适用房36万平方米以上，新增廉租住房保障对象1700户。二是继续实施村寨防火改造工程，确保年内完成423个村寨的改造任务。三是实施北部三县少数民族村寨房屋火灾统保工程。按照“政府主导、财政扶持、市场运作、自愿参保”的原则，以政府投入为主、居民出资为辅，对北部三县少数民族村寨11.7万座木楼统一进行火灾保险，因火灾毁坏的木楼每座将获得2万元保险赔偿，逐步建立村寨房产防火安全保障机制，有效解决村寨居民因火灾致贫和返贫问题。四是实施巷道疏通工程。清理巷道违法建筑，整治巷道卫生环境，逐步完善巷道交通、排水和路灯设施，通过打通支线和下穿节点，实现主次干道与小街小巷互联互通，进一步改善市民出行状况。

创建平安柳州，维护社会稳定。深化平安柳州建设，不断完善社会治安打防控体系，依法打击各种形式的犯罪活动、恐怖破坏活动和黑恶势力活动。巩固公安“三基”工程和科技强警建设成果，加快“天网工程”二期建设。积极推进公安资源整合。深入实施社区和农村警务战略，构建平安和谐小区。继续开展领导干部大接访和专项下访活动，加强信访和“三大纠纷”调处，努力维护社会稳定。高度重视安全生产。加强食品药品监管，逐步建立食品药品安全责任体系。

八、繁荣发展社会各项事业，促进社会和谐

统筹推进社会事业发展，加强文化建设，不断满足人民群众日益增长的社会公共服务和精神文化需求，促进社会和谐发展。

优先发展教育事业。巩固发展“两基”成果，大力推进素质教育，加快义务教育均衡发展。整合城中片区教育资源，合理调整中小学校布局。大力发展职业教育，建成职教园一期并在秋季启用；积极争创首个国家民族地区职业教育综合改革实验区，着力打造西南一流的职业教育基地。不断完善教育助学体系。全面落实农村义务教育经费保障和城市义务教育免杂费政策，确保财政公用经费满足学校运转需要。利用国家扶持西部特殊教育学校政策，建立健全特殊教育体系。加快农村寄宿制小学建设，对无条件发展农村寄宿学校的偏远山区适龄就学儿童，开展接到乡镇学校集中上学试点。着力解决农村留守儿童教育和外来务工人员子女教育问题。扩大农村学前教育规模，推动乡镇中心幼儿园建设。根据国家和自治区的部署，逐步提高教师收入水平。

积极构建和谐文化。加快历史名城和文化名城建设，积极推动白莲洞古人类生态园、工业历史博物馆、军事博物园、水上流动舞台等文化重点项目建设，促进文化产业发展。稳妥推进文化体制改革，提高城市文化竞争力。推进精品剧目创作。合理利用文博和非物质文化资源，扎实抓好第三次全国文物普查工作，加强文化遗产传承、保护和利用，提升城市历史文化内涵。广泛开展人民群众喜闻乐见的文化活动。不断完善公共文化服务体系，实施“文化惠民”工程，力争年内实现各县区全部建有图书馆和国家文化信息资源共享工程支中心，建设40个城乡基层文化活动点、400个农家书屋，解决2257个边远山区村屯和一批市郊村屯的广播电视信号接收问题。

加快卫生、人口计生和体育等事业发展。启动“国家卫生城市”创建工作。按照国家统一部署，推进医疗卫生体制改革。加大农村和社区医疗卫生基础设施建设力度，促进基本公共卫生服务均等化，建设41个乡镇卫生院，扩建六县11家县级医院；建成120个甲级村卫生所，实现全市所有行政村都有甲级卫生所的目标。加强艾滋病、结核病等重大疾病防控。继续推进新农合工作，确保全市参合率达到90%。加强人口与计生基层基础工作，完善稳定低生育水平工作机制，开展争创全国、全区计划生育优质服务县区活动。全面发展体育事业，完善公共体育设施；充分利用“百里柳江”水上资源，积极策划、大力发展水上运动项目。

加强精神文明建设。大力推进社会主义核心价值体系建设。积极开展建国60周年庆祝活动。弘扬新时期爱国主义精神和以改革创新为核心的创新精神，加强理想信念教育，倡导爱国主义、集体主义、社会主义思想。加强社会公德、职业道德、家庭美德教育，创新未成年人思想道德建设工作。全面落实妇女儿童发展纲要。以创建文明城市为载体，大力开展文明单位、文明社区、文明村镇的创建工作，形成全民参与精神文明创建的良好格局。加强国防教育和国防后备力量建设，巩固双拥共建成果。保障少数民族合法权益，巩固和发展平等团结互助和谐的社会主义民族关系。加大“真实柳州、精彩柳州”宣传力度，进一步提升城市形象。

九、加强政府自身建设，努力提高政府工作水平

深入开展学习实践科学发展观活动，进一步增强应对危机、把握机遇、加快发展的能力，切实履行政府职责，更好地为人民群众服务。

进一步优化政务环境。加强各级政务服务中心建设。深化行政审批制度改革，努力实现行政审批时限的再提速。坚持市县两级重大行政决策听证制度，积极推进信息公开，让广大群众更好地行使知情权、参与权和监督权。加快电子政务的推广应用，积极推进网上审批，提高电子政务的公共服务能力。继续抓好经济普查工作。

加强民主法治建设。认真落实市委《关于进一步加强和改进人大工作的意见》。各级政府要自觉接受同级人大及其常委会的监督，切实加强与政协的联系，认真听取民主党派、工商联、无党派人士和各人民团体的意见，进一步提高决策的科学化、民主化。深入开展“五五”普法，积极开展法律援助，全面推进依法治市。积极推进厂务公开，完善村务公开，不断健全基层群众依法自治机制。

加强党风廉政建设。完善绩效考评制度，认真开展绩效考评工作。建立健全惩治和预防腐败体系，落实党风廉政建设责任制，加强领导干部和国有企业领导人员廉洁自律工作，认真抓好农村基层党风廉政建设。开展纠风专项治理，建立健全防治不正之风的长效机制。加强审计监督工作，不断提高审计质量和水平。

各位代表，今年我们面临的形势非常严峻，发展的任务相当繁重，肩负的责任十分重大。让我们以邓小平理论和“三个代表”重要思想为指导，深入贯彻落实科学发展观，充分发扬改革创新精神，在市委的坚强领导下，坚定信心，奋勇向前，攻坚克难，努力保持经济社会平稳健康较快发展，以优异的成绩迎接新中国成立60周年！
